# A Comparative Analysis of Double-J Stent and Nephrostomy With a Transanastomotic Stent in Open Pyeloplasty for Pelviureteric Junction Obstruction in Children: A Prospective Observational Study

**DOI:** 10.7759/cureus.90035

**Published:** 2025-08-13

**Authors:** Ankit Kasundra, Suresh Thanneeru, Reyaz Ahmad, Zainab Ahmad, Amit Gupta, Roshan Chanchalani, Pramod K Sharma

**Affiliations:** 1 Department of Pediatric Surgery, All India Institute of Medical Sciences, Bhopal, Bhopal, IND; 2 Department of Anesthesiology, All India Institute of Medical Sciences, Bhopal, Bhopal, IND

**Keywords:** double j stent, hydronephrosis, pelviureteric junction obstruction, transanastomotic stent, ureteropelvic junction obstruction

## Abstract

Background

This study aims to compare the outcomes of double-J (DJ) stenting versus nephrostomy with transanastomotic (NTA) stenting in children undergoing open pyeloplasty, focusing on operative time, complications, postoperative pain, hospital stay, cost-effectiveness, and caregiver satisfaction.

Methodology

A prospective, observational study was conducted on 30 children who underwent open pyeloplasty between January 2023 and June 2024. Intraoperative and postoperative parameters were analyzed, including postoperative pain, complications, hospital stay, treatment cost, caregiver satisfaction scores, and postoperative renal function at the six-month follow-up.

Results

The DJ stent group (Group A) had a significantly shorter operative time (118 minutes vs. 160 minutes, p = 0.0001), lower postoperative pain from 24 to 72 hours (p < 0.05), and higher caregiver satisfaction (8.76 vs. 6.56, p = 0.001). Although the NTA group (Group B) experienced fewer complications, the difference was insignificant (p = 0.45). However, NTA stents were easier to remove (not requiring a second anesthesia) and were more cost-effective (p < 0.0001). No significant difference was observed in postoperative renal function between the groups.

Conclusions

This study highlights that DJ stenting offers better pain control, shorter hospital stay, and higher caregiver satisfaction. NTA stenting may be considered in specific scenarios such as failed DJ stent placement, financial constraints, or large hydronephrotic kidneys where nephropexy is advantageous.

## Introduction

Pelviureteric junction (PUJ) obstruction, occurring in approximately 1 in 5,000 live births, often requires surgical correction via dismembered pyeloplasty. Using a stent remains a matter of choice, depending on the surgeon’s experience, expertise, and patient-specific characteristics. When appropriate stents are used, adequate drainage of the surgical site is ensured, making the procedure reproducible even by fellows and trainees. Various types of stents are available for drainage. Double-J (DJ) stents allow for better drainage and earlier hospital discharge but may necessitate additional imaging to confirm stent position. DJ stents can also cause stent-related complications and typically require readmission and general anesthesia for removal [1,2].

Alternatively, nephrostomy with a transanastomotic (NTA) stent is widely available, does not require imaging to confirm placement, and is useful when a DJ stent cannot be negotiated into the bladder. The external stent can be removed at the bedside, and a nephrostogram can be performed if needed. However, due to the presence of external drains, there is a higher risk of infection and increased postoperative pain. Therefore, this study compared DJ stents and NTA with nephrostomy in open pyeloplasty concerning operative time, intraoperative and postoperative complications, postoperative pain, hospital stay, cost-effectiveness, and caregiver satisfaction.

## Materials and methods

Study design

This prospective, observational cohort study was conducted in the Department of Pediatric Surgery at a tertiary care center from January 2023 to June 2024. Ethical approval was obtained from the Institutional Human Ethics Committee (IHEC-PGR/2000 DM-MCh/Jan 04, dated September 12, 2022).

Inclusion and exclusion criteria

Children under 15 years of age diagnosed with PUJ obstruction requiring pyeloplasty, including redo pyeloplasty, bilateral obstruction, and those initially managed with percutaneous nephrostomy (PCN), were included. Exclusion criteria were patients managed via laparoscopy/retroperitoneoscopy, those with renal anomalies (e.g., horseshoe kidney, pelvic kidney), and families who declined consent.

Data collection

Demographic data, laterality of the pathology, presentation, and complications were recorded. The surgery and stent type were determined by the operating surgeon based on the patient’s characteristics, regardless of the study. Patients were divided into the following two groups: DJ stent (Group A) and NTA (Group B). All children received general anesthesia with a caudal epidural block using 0.2% bupivacaine. An open pyeloplasty with a flank or subcostal approach was performed. In Group A, an appropriately sized DJ stent was inserted into the bladder. In Group B, a 5 Fr infant feeding tube was passed into the mid-ureter across the anastomosis, and a 6 Fr Malecot’s catheter was inserted into the lower calyx. The nephrostomy tube was left for drainage, and the transanastomotic tube was closed and covered with a dressing.

As the choice of stenting technique was solely based on the operating surgeon’s preference and not on a protocol, a formal sample size calculation was not performed. Instead, we included all consecutive patients who underwent pyeloplasty with either technique during the study period. Intraoperative details (e.g., operating time, complications, etc.) were recorded. At the end of surgery, each patient received IV paracetamol at 15 mg/kg.

Postoperative care and pain assessment

Post-operatively, IV Paracetamol at a dose of 15 mg/kg every 6 hours was administered as an analgesic. Pain was assessed using age-appropriate scales: Face, Legs, Activity, Cry and Consolability (FLACC) scale for 2 months to 5 years, Wong-Baker FACES® Pain Rating Scale for 6 to 8 years, and the Numeric Rating Scale (NRS) for children over 8 years). The NRS and FLACC Scale are freely available for use in both research and clinical settings without licensing restrictions. Necessary permission was obtained to use the Wong-Baker FACES® Pain Rating Scale. Pain assessments were conducted at 0, 3, 6, 12, 24, 48, and 72 hours post-surgery. If scores were over 3, non-pharmacological measures were implemented to comfort the child; if scores remained above 3, rescue analgesia was provided in the form of IV Fentanyl 1-3 μg/kg or Ibuprofen 10 mg/kg if the patient could take oral medication.

All patients were allowed oral intake on the evening of surgery. Foley’s catheter was removed within 24-48 hours in both groups. In Group A, patients were discharged after the perinephric drain was removed. The DJ stent was removed under general anesthesia after four to six weeks. In Group B, the transanastomotic tube was removed after 48-72 hours, and the nephrostomy tube was gradually raised with output monitoring. The nephrostomy was clamped once the output decreased. It was removed if the patient was asymptomatic after 24 hours of clamping and minimal output in the perinephric drain. Patients were discharged only after all drains were removed.

Catheter, drain, and stent removal times were documented. Stent-related events such as dysuria, frequency, and hematuria were recorded. Patient satisfaction was evaluated using the NRS from 1 to 10, where 1 indicated complete dissatisfaction and 10 reflected maximum satisfaction. The total hospital stays and costs were calculated for the current admission and the admission needed for stent removal, starting from the day of surgery. It is possible that some patients might be admitted early for registration in health schemes. The overall cost was determined based on the standard charges at our center, including charges for admission and bed, surgical procedures, investigations, and consumables.

Outcome measures

Catheter, drain, and stent removal times were documented. Stent-related events such as dysuria, frequency, and hematuria were noted. Patient satisfaction was assessed using the NRS (1 to 10), where 1 indicated complete dissatisfaction and 10 indicated maximum satisfaction. Total hospital stay and cost were calculated for both the index admission and the readmission for stent removal, starting from the day of surgery. Early admissions for registration in health schemes were accounted for. Overall cost included standard institutional charges for admission, bed occupancy, surgery, investigations, and consumables.

Follow-up

Patients were followed up at three months with ultrasonography (USG) and at six months with USG and a dynamic renogram. Data confidentiality was maintained, and only the investigators accessed the patient information.

Statistical analysis

Data were analyzed using SPSS version 27 (IBM Corp., Armonk, NY, USA). Quantitative data were expressed as mean ± standard deviation. Student’s t-test was applied; for non-parametric variables, the Mann-Whitney U test was used. Categorical variables were analyzed using the chi-square test. A p-value <0.05 was considered statistically significant.

## Results

**Figure 1:**
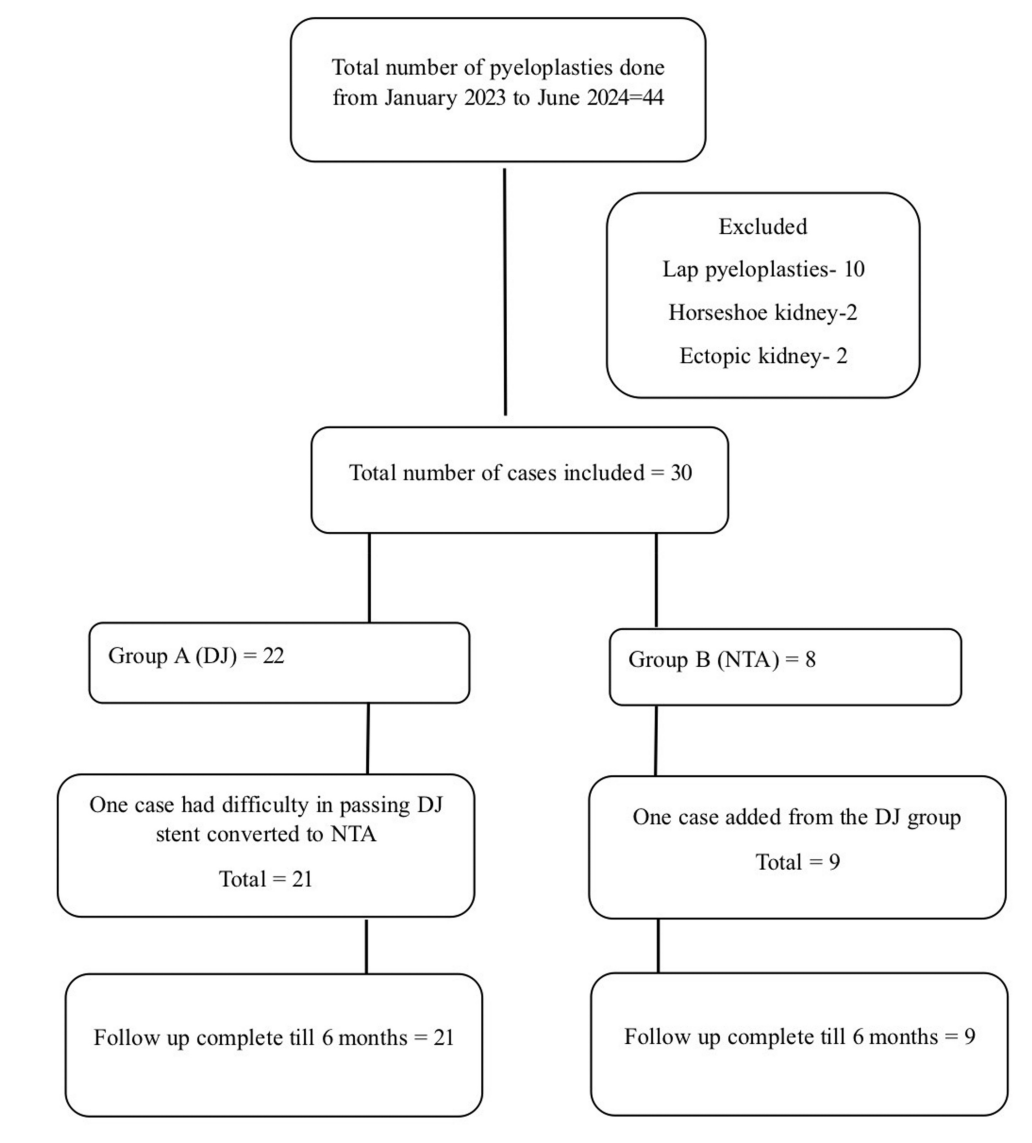
Consort flow diagram depicting patient enrollment and allocation. DJ = double J; NTA = nephrostomy with transanastomotic

A total of 30 patients were enrolled in this study, with 21 patients in Group A and nine in Group B. Follow-up was completed for all participants. Figure 1 provides details regarding patient recruitment and follow-up.

The mean age of patients in Group A was 54.29 ± 56.50 months (range = 2 to 168 months), and in Group B, it was 20.55 ± 26.26 months (range = 2 to 72 months). There were 24 males and six females in the cohort. Symptomatic presentation was more common than incidental or antenatal detection; however, there was no statistically significant difference between the groups regarding clinical presentation or laterality (Table 1).

**Table 1:** Baseline characteristics and intraoperative data. *: P-values <0.05 statistically significant by Mann-Whitney test/unpaired t-test or chi-square test.

Parameters	Group A (n = 21)	Group B (n = 9	Effect size/df	P-value
Age in months	54.29 ± 56.50	20.55 ± 26.26	-	0.14
Gender			0.21/1	0.39
Male	18 (85.7%)	6 (66.7%)		
Female	3 (14.2%)	3 (33.3%)		
Presenting symptoms (children had more than one presenting symptom)			0.11/1	0.62
Antenatally detected	9 (42.8%)	4 (19%)		
Pain	10 (47.6%)	5 (55.5%)		
Incidentally found	9 (42.8%)	3 (33.3%)		
Urinary tract infection	9 (42.8%)	5 (55.5%)		
Palpable mass	9 (42.8%)	5 (55.5%)		
Laterality of pathology			0.71/1	0.07
Left	3 (14.2%)	4 (19%)		
Right	18 (85.7%)	5 (55.5%)		
Intraoperative data				
Type of incision			0.18/1	0.42
Flank	16 (76.1%)	8 (88.8%)		
Sub-costal	5 (23.8%)	1 (11.1%)		
Length of incision (mean in cm)	3.88 ± 0.73	3.22 ± 0.44*	-	0.02*
Difficulty in passing stent	3 (14.2%)	0 (0%)	0.10/1	0.73
Type of drain			0.23/1	0.30
Closed redivac drain	15 (71.4%)	8 (88.8%)		
Soft drain	6 (28.5%)	1 (11.1%)		
Operative time (mean in minutes)	118.52 ± 21.47	160.33 ± 17.55	-	0.0001*

The average anteroposterior pelvic diameter (APPD) of the renal pelvis on the affected side was 3.75 ± 2.13 cm in Group A and 4.63 ± 2.45 cm in Group B (p = 0.10). A total of 16 patients underwent micturating cystourethrogram (MCUG) for various indications. Vesicoureteral reflux (VUR) was identified in only one patient in Group A. One patient in each group underwent a preoperative PCN for suspected pyonephrosis. The mean differential renal function of the affected kidneys was 38.78 ± 13.25 (range = 14-59) in Group A and 41.01 ± 20.08 (range = 15-73) in Group B (p = 0.72). Both groups were comparable in preoperative evaluation, with no significant differences observed. There was no difference between the groups regarding the type of surgical incision or drain used (p > 0.05). Difficulty in negotiating the stent occurred in three (14.28%) patients in Group A but not in Group B (p > 0.05). Group A had a significantly shorter incision length and operative time than Group B (p < 0.05). For the first 12 hours postoperatively, there was no significant difference in pain scores between the groups. However, at 24 hours and beyond, Group B exhibited significantly higher pain scores (p < 0.05). Breakthrough pain episodes, defined as pain occurring despite routine analgesia and requiring rescue analgesia, were more frequent in Group B (p < 0.05) (Table 2).

**Table 2:** Comparison of postoperative pain score between the study groups. ^+^: P-values <0.05 compared to baseline zero-hour data by intragroup comparison using the repeated analysis of variance test; *: P-values <0.05 compared to DJ vs. NTA value by intergroup comparison using the Mann-Whitney U test. DJ = double J; NTA = nephrostomy with transanastomotic

Time duration	Group A (n = 21)	Group B (n = 9)	P-value
0 hours	1.90 ± 0.62	2.11 ± 0.33	0.54
6 hours	1.66 ± 0.80	2.00 ± 0.05	0.14
12 hours	1.57 ± 0.81	1.88 ± 0.60	0.23
24 hours	0.85 ± 0.73^+^	2.00 ± 0.05	0.0006*
48 hours	0.95 ± 0.80^+^	2.11 ± 0.33	0.001*
72 hours	0.52 ± 0.75^+^	1.44 ± 0.88^+^	0.021*
Intragroup P-value	<0.001	0.04	-
Breakthrough pain episodes			
Number of patients	7	3	-
Number of episodes	15	3	-
Severity of breakthrough pain			
Moderate	12	3	-
Severe	3	0	-

In terms of complications, two patients in Group A experienced bladder spasms, and six developed hematuria, which improved with oral oxybutynin at a dose of 0.2 mg/kg in two divided doses. Three patients developed febrile urinary tract infections (UTIs) after discharge: one in Group A and two in Group B. All three required hospital admission and antibiotic treatment based on culture and sensitivity reports. One patient in Group A experienced worsening hydronephrosis postoperatively and underwent redo pyeloplasty three months after the initial surgery. In Group B, one patient was found to have vesicoureteric junction (VUJ) obstruction on postoperative nephrostogram and subsequently underwent ureteric reimplantation. Overall, the incidence of complications was not significantly different between the two groups. The mean drain dwell time was considerably shorter in Group A (p < 0.05), whereas the stent dwell time was significantly shorter in Group B (p < 0.05). The mean PCN dwell time in Group B was 8.42 ± 5.06 days (Table 3).

**Table 3:** Comparison of postoperative data in study groups. *: P-values <0.05 statistically significant by the Mann-Whitney test/unpaired t-test or chi-square test. UTI = urinary tract infection

Parameters	Group A (n = 21)	Group B (n = 9)	Effect size/df	P-value
Postoperative complications			0.34/2	0.453
UTI	01	02		
Bladder spasm	02	00		
Hematuria	06	00		
Dwelling time of catheters and stents				
Mean drain dwelling time (days)	3.62 ± 0.97	5.77 ± 0.97	-	0.0003*
Mean stent dwelling time (days)	35 ± 5.15	4.0 ± 0.81	-	<0.0001*
Healthcare outcome metrics				
Caregiver/Parent satisfaction score	8.76 ± 1.04	6.56 ± 0.72	0.70/1	0.0001*
Readmission	23	03		0.04*
Hospital stays (days)	8.62 ± 1.71	12.33 ± 2.35	-	0.0007*
Overall cost (Indian rupees)	9,077.62 ± 59.88	7,912.88 ± 129.56		<0.0001*

Postoperative outcomes related to renal parameters were comparable in the two groups, with no statistically significant differences. Both groups demonstrated a significant reduction in renal size and APPD of the renal pelvis (p < 0.05). Improvement was also observed in postoperative differential renal function (DRF) and drainage; however, the increase in differential function did not reach statistical significance. Group A showed a higher rate of readmission but a significantly shorter total hospital stay (p < 0.05). Parental satisfaction was significantly higher in Group A (p < 0.05), while the overall treatment cost was significantly lower in Group B (p < 0.05) (Table 4).

**Table 4:** Renal outcomes of both groups. APPD = anteroposterior pelvic diameter

Outcomes	Group A (N = 21)			Group B (N = 9)		
	Before	After	P-value	Before	After	P-value
Kidney size (in cm)	9.24 ± 2.81	8.22 ± 2.01	0.005	10.21 ± 5.18	8.88 ± 4.71	0.01
APPD (in cm)	3.75 ± 2.13	2.30 ± 1.52	0.001	4.27 ± 2.25	2.30 ± 1.29	<0.01
Differential renal function	38.78 ± 13.25	40.17 ± 9.79	0.75	41.01 ± 20.08	38.28 ± 14.23	0.4

## Discussion

The use of stents in pyeloplasty has been a subject of debate. It is believed that, with appropriate stents, proper drainage of the repair is ensured, making the procedure reproducible and leading to early discharge from the hospital. This study compares DJ stents and nephrostomy with a transanastomotic stent in pediatric pyeloplasty for PUJ obstruction.

The mean age of the children was slightly higher in Group A, and there was a predominance of male children in both groups. Obstructive symptoms, such as pain and a palpable mass, were the most common presentations, rather than antenatal detection. The left kidney was more frequently affected, and no cases of bilateral involvement were reported. No significant difference was observed between the groups regarding APPD of the renal pelvis and differential renal function. The incidence of VUR could not be accurately determined, as only a subset of patients underwent MCUGs, with just one case of reflux detected out of 16 MCUGs performed, which is much lower than the incidence reported by Bomalaski et al. (18.30%) [3].

The average operating time was notably shorter in Group A compared to Group B. This may be because the surgeon selected NTA procedures for more complex cases. The reported operating time in our study was 118 minutes in Group A, comparable to Elmalik et al. [1] (119.7 ± 3.8 minutes) and much shorter compared to Sarhan et al. [4] (145 ± 18 minutes), Lee et al. [5] (140 ± 46 minutes), and Braga et al. [6] (164 minutes). However, our operating time in Group A was longer than that of Nagdeve et al. [2] (78.9 ± 8.17 minutes). The operating time in Group B was comparable to other studies with external stents. Sarhan et al. [4] reported 150 ± 20 minutes for pyeloureteral stents, Lee et al. [5] reported 147 ± 35 minutes in the externalized uretero-pyelostomy stent group, and Braga et al. [6] reported 164 minutes for the Salle stent group. The challenges encountered during stent negotiation were 14.28% in our study, while Nagdeve et al. [2] reported 21.7% of the cases, and Wang et al. [7] reported 6.9% of the cases. This highlights the real-time issue of passing DJ stents during pyeloplasty.

Preoperative APPD and DRF were comparable between groups, consistent with findings from Bansal et al. [8] and Chertin et al. [9], who observed no significant baseline anatomical or functional differences between patients undergoing open or laparoscopic pyeloplasty. VUR was infrequent in our cohort, aligning with its reported low prevalence in isolated PUJ obstruction [10,11].

The length of the incision, which could be an indirect factor influencing postoperative pain, was significantly shorter in Group A compared to Group B. This may be because the surgeon prefers to make a slightly larger incision, anticipating that these kidneys are already severely affected and complications are expected in advance. The study observed a significant difference in pain scores and the number of breakthrough pain episodes between Group B and Group A. This difference might be related to the number of catheters or drains and the incision length, as mentioned earlier. Initially, similar pain scores up to 24 hours may be due to the effective nerve blockade administered by the anesthetist before the procedure. No other studies involving alternative stenting techniques have investigated pain scores, making our study the first to compare pain scores across different stenting techniques. However, studies with the DJ stent vs. no stenting have evaluated pain scores similar to Nagdeve et al. [2], where the DJ group received more doses than the no-stent group.

Regarding complications, UTI and hematuria were more common in Group A, likely due to the prolonged presence of a foreign body in the urinary tract. The complication rate observed with the DJ stenting in this study was high compared to Elmalik et al. [1] (15.5%), Sarhan et al. [4] (8%), Braga et al. [6] (9.9%), and Lee et al. [5] (5.2%) but lower compared to Garg et al. [12] (95%). The complications of Group B observed in this study (22.22%) were comparable to Lee et al. [8], who reported 20.8% complications in externalized stents. Among the reported complications across the studies, UTI and stent migration are the most frequent.

None of the patients in this study had a leak or prolonged drain output. The mean drain removal time was 3.62 ± 0.97 days in Group A compared to 5.71 ± 0.95 days in Group B, which was significantly lower in Group A (p = 0.001). There were no reports of stent fracture or migration in this study. All patients were catheterized intraoperatively, which was removed after 24 to 48 hours. The stent dwelling times differed from other studies, with Sarhan et al. [4] (42 days for DJ, 9 days for externalized stent), Castagnetti et al. [13] (7-9 days for externalized stent), Garg et al. [12] (14 days for DJ, 7 days for nephrostomy removal), Zaidi et al. [14] (10 days for externalized stent removal). The DJ stent removal period varied across studies from two weeks to six weeks; in this study, the DJ dwelling time is approximately five weeks. The average transanastomotic stent removal time in Group B cases was around four days, shorter than other studies involving externalized stents.

Group A exhibited significantly higher readmission rates than Group B, mainly due to the necessity of DJ stent removal. Additionally, two patients in the DJ group were re-admitted for complications such as UTI and recurrence. In Group B, two children were readmitted with UTI, and one was admitted with suspected VUJ obstruction, which was later confirmed by a nephrostogram and required ureteric reimplantation.

The overall hospital stay was significantly shorter in Group A, even after including readmission for stent removal (8.62 ± 1.71 days) compared to Group B (12.14 ± 2.41 days, p = 0.0007). Hospital stays in our study were longer than those reported by Arda et al. [15] (5.3 ± 2 days), Elmalik et al. [1] (2.71 ± 0.25 days), Nagdeve et al. [2] (4.28 ± 0.67 days), Sarhan et al. [4] (3.7 days), Braga et al. [6], Lee et al. [5] (1.9 ± 1.6 days), Garg et al. [12] (5.5 ± 4.79 days), and Kong et al. [16], likely because DJ stent removal days were included in the total hospital stay in our study. The hospital stay in Group B was comparable to that of Garg et al. [12] (11.95 ± 1.395 days) but was longer than other studies [1,9,14] using externalized stents. The extended stay in this study was affected by social factors, as many patients travelled long distances and were discharged only after all drains and tubes were removed.

The overall cost per patient was significantly higher in Group A (₹9,077.62 ± 59.88) compared to Group B (₹7,912.71 ± 146.50) (p < 0.0001). However, the total cost in this study was significantly lower than in Garg et al. [12] and Braga et al. [6]. This is likely due to lower hospital care charges in government institutions, but the stent cost per se was much lower than in other studies.

This study was unique in its assessment of patient satisfaction scores, which found higher scores in Group A than in Group B, likely due to lower pain, fewer tubes and drains, and shorter hospital stays.

Both groups showed significant improvement in renal pelvis APPD. Still, the improvement in differential renal function was less pronounced in Group B, possibly due to preoperative supra-normal functioning kidneys, which exhibited falsely increased preoperatively and normal function postoperatively. A longer follow-up may reveal clearer results. Overall, renal function was comparable between the groups. There was no recurrence in Group B, whereas one recurrence occurred in Group A, requiring redo-pyeloplasty, and the patient is now under regular follow-up.

This study reported a lower recurrence rate (0.48% in Group A and none in Group B). The reported recurrences across various studies include Braga et al. [6] (5% DJ, 5.3% Salle), Kong et al. [16] (8.3% DJ, 11.1% externalized stent), Lee et al. [5] (2.63% DJ, none in externalized stent) and Sarhan et al. [4] (4.54% DJ, 3.07% externalized stent).

Overall, this study found shorter operative times, less postoperative pain, higher caregiver satisfaction, and shorter stays, though at higher costs, in Group A, while Group B experienced fewer complications and easier stent removal during the same admission without anesthesia. We recommend routine DJ stent use due to better pain management, satisfaction, and trainee exposure for cystoscopies during removal, reserving transanastomotic stenting with nephrostomy for cases where DJ stent negotiation is difficult, aiming to optimize cost-effectiveness and address the limited availability of anesthesia. Nephrostomy also assists nephropexy in large, sagging kidneys prone to kinking, preventing anastomotic recurrence in PUJ obstruction.

Limitations

This study was limited by its small sample size and non-randomized design, which may restrict the generalizability of findings. Furthermore, a longer follow-up is necessary to fully assess long-term renal function outcomes.

## Conclusions

In stented pyeloplasty for PUJ obstruction, DJ stents are associated with better postoperative pain scores and higher parental satisfaction. The NTA stents should be reserved for cases where DJ stent placement is not feasible or in patients with large, severely hydronephrotic kidneys.
